# Emergence of overt myeloma in a patient with chronic lymphocytic leukemia on ibrutinib therapy

**DOI:** 10.1002/ccr3.3019

**Published:** 2020-07-15

**Authors:** Ayad M. Al‐Katib, Hussein Gaith, Dahlia Sano, Sayf Al‐Katib, Michelle Bonnett, Zyad Kafri

**Affiliations:** ^1^ Lymphoma Research Laboratory Department of Internal Medicine Wayne State University School of Medicine (WSU SOM) Detroit MI USA; ^2^ Department of Oncology Karmanos Cancer Institute WSU SOM Detroit MI USA; ^3^ Department of Diagnostic Radiology and Molecular Imaging Beaumont Health Oakland University William Beaumont School of Medicine Royal Oak MI USA; ^4^ Department of Pathology Ascension St. John Hospital and Medical Center Detroit MI USA; ^5^ Van Elslander Cancer Center Grosse Pointe Woods MI USA

**Keywords:** bruton tyrosine kinase inhibitors, chronic lymphocytic leukemia, ibrutinib, multiple myeloma

## Abstract

Ibrutinib is approved for chronic lymphocytic leukemia (CLL). However, its role in the treatment of multiple myeloma (MM) is not clear and is under investigation. We report a case of CLL that developed MM while on therapy with ibrutinib indicating that this drug may not be active against MM.

## Graphical abstract

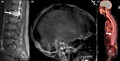

## 1 INTRODUCTION

Chronic lymphocytic leukemia is the most common leukemia in the United States of America. It affects mainly elderly males above the age of 70. Diagnosis of CLL requires the presence of lymphocytosis (>5.0 × 10^9^/L) of monoclonal B cells and characteristic immunophenotype in the peripheral blood or bone marrow.^1^, ^2^, ^3^ Chemoimmunotherapy consisting of cytotoxic chemotherapy agents with anti‐CD20 monoclonal antibody rituximab, Bruton tyrosine kinase (BTK) inhibitors like ibrutinib and BCL‐2 inhibitors (venetoclax) are all considered first‐line treatment options for patients with CLL depending on their clinical and genetic factors.^2^

According to cancer statistics 2020, MM will account for 18% of hematological malignancies (32 270 cases of 178 520).^4^ It is more common in males around the age of 65 years and in African Americans compared with Caucasians. According to current International Myeloma Working Group (IMWG) criteria, diagnosis of MM depends on the presence of, and number of clonal plasma cells in the bone marrow or a biopsy‐proven plasmacytoma, immunoglobulin light chain restriction and ratio, skeletal lesions detected by MRI, and presence of end‐organ damage (hypercalcemia, renal failure, anemia, and bone lesions), collectively known as SLiM CRAB criteria.^5^ Management of MM includes immune modulators (like thalidomide, or lenalidomide), proteasome inhibitors like bortezomib and corticosteroids (dexamethasone). Newer drugs are used as single agents or with various combinations including carfilzomib, pomalidomide, daratumumab, ixazomib, elotuzumab, and selinexor.^6^

The coexistence of both CLL and MM is very rare.^7^ A study done between 2000 and 2015 at Mayo Clinic showed 28 of 10,735 (0.26%) patients diagnosed with MM to also have CLL.^1^ As summarized in Table S1, 15 patients (53%) of those were diagnosed with CLL/SLL before MM; 11 patients (39%) were diagnosed simultaneously, and 2 patients developed CLL after MM diagnosis (8%). The clonal relationship between CLL and MM when they codevelop in the same patient is not clearly defined with some studies indicate two separate clones^8^, ^9^ while others support a common origin.^10^, ^11^ However, the ability of ibrutinib therapy to prevent development of MM in CLL patients is not known. Here, we report a patient with established diagnosis of CLL who developed MM while on maintenance therapy with Ibrutinib.

## 2 CASE PRESENTATION

A 55‐years old Caucasian male with past medical history of Hashimoto's disease, pernicious anemia, vitamin D deficiency, vitiligo, low testosterone, and cluster headaches presented to his primary care physician in 2013 for routine visit without new complaints. He was found to have mild elevation of white blood cell (WBC) count (14.1 × 10^3^/µL, upper limit of normal (ULN): 10.6 × 10^3^/µL). His WBC differential count showed normal neutrophil count of 3.1 × 10^3^/µL, elevated lymphocyte count of 10.2 × 10^3^/µL (ULN: 3.8 × 10^3^/µL), normal hemoglobin of 13.8 gm/dL, and normal platelets of 201 (×10^3^/µL). Review of blood smear showed small lymphocytes with clumped chromatin characteristic of CLL. Physical examination revealed bilateral mid‐cervical lymph nodes of 1‐1.5 cm, a left supraclavicular lymph node (~1.5 cm), and a right axillary lymph node (~1‐1.5 cm). There was no hepatosplenomegaly. Flow cytometry revealed monotypic B‐cell population expressing dim kappa light chain, CD5, CD19, CD20, and CD23 consistent with CLL. Cells were negative for CD10 and FMC7. Cytogenetics by fluorescence in situ hybridization (FISH) showed 11q deletion in 77% of the nuclei and deletion of chromosome 13 in 43.5% of the nuclei. Additional work up at diagnosis included immunoelectrophoresis (IEP) that showed an IgA kappa monoclonal gammopathy of 75 mg/dL and a possible faint IgM heavy chain restriction. His serum‐free Kappa light chain (FLC) was elevated at 3.15 mg/dL (normal: 0.33‐1.94), lambda FLC was normal (0.96 mg/dL, normal range: 0.57‐2.63 mg/dL). The patient was diagnosed with stage I CLL by Rai classification with two risk factors, a CD38 > 30%, and ZAP 70 > 30%.^12^

**FIGURE 1:**
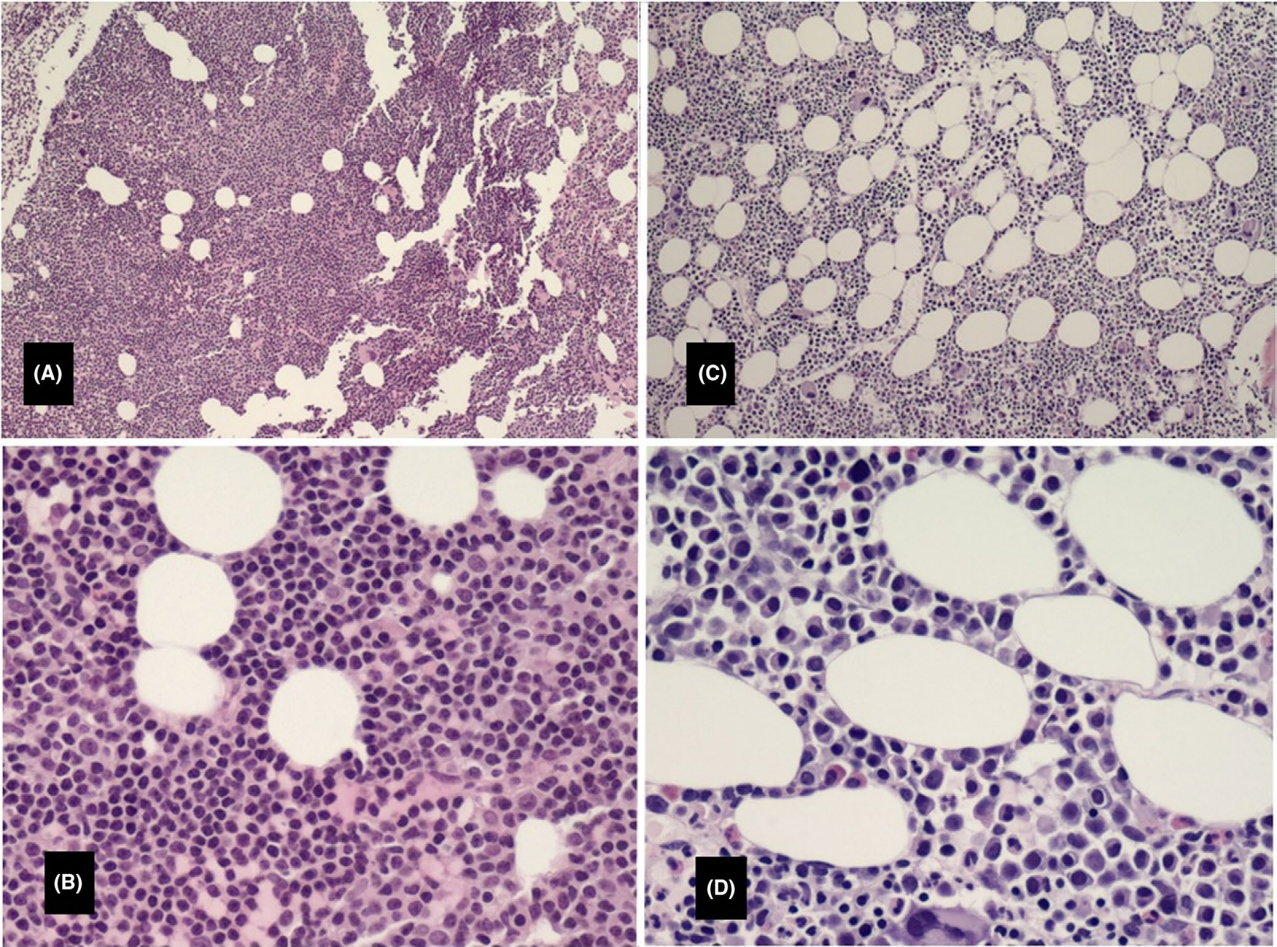
A, Bone marrow biopsy section showing diffuse cellular infiltration (10×), B, Higher magnification (40×) of the same bone marrow biopsy section as in (A) showing the cellular infiltration as monotonous small lymphocytes typical of CLL/SLL. C and D, Bone marrow biopsy with Plasma cell myeloma at 10× and 40×, respectively

The patient was under observation since he had no indication for CLL therapy until two and a half years later (2016) when he developed progressive fatigue and decreased blood counts (platelets 115 × 10^3^/µL, hemoglobin 11 gm/dL, and neutrophils 1.2 × 10^3^/µL). His total WBC also increased (26.2 × 10^3^/µL) as well as the size of his lymph nodes. A bone marrow aspiration and biopsy (Figure 1A) at that time showed increased cellularity estimated at 90%. Approximately 80%‐90% of the cellularity was composed of a diffuse infiltrate of small lymphocytes (Figure 1B). The remaining cellular elements were adequate‐appearing megakaryocytes, maturing erythroid and myelocytic cells; there was no increase in the number of plasma cells. FISH panel showed deletion of 11q in 87% of nuclei and a deletion of 13q in 14.5% of nuclei indicating persistence of same abnormal clones found at diagnosis. The patient was started on therapy with ibrutinib (560 mg orally once a day) and rituximab (375 mg/m^2^ iv on day 1 of every 28 days‐cycle) and tolerated therapy well. He achieved good clinical response and completed 6 cycles of treatment. The patient was put on ibrutinib maintenance dose (420 mg daily) without any side effects.

**FIGURE 2:**
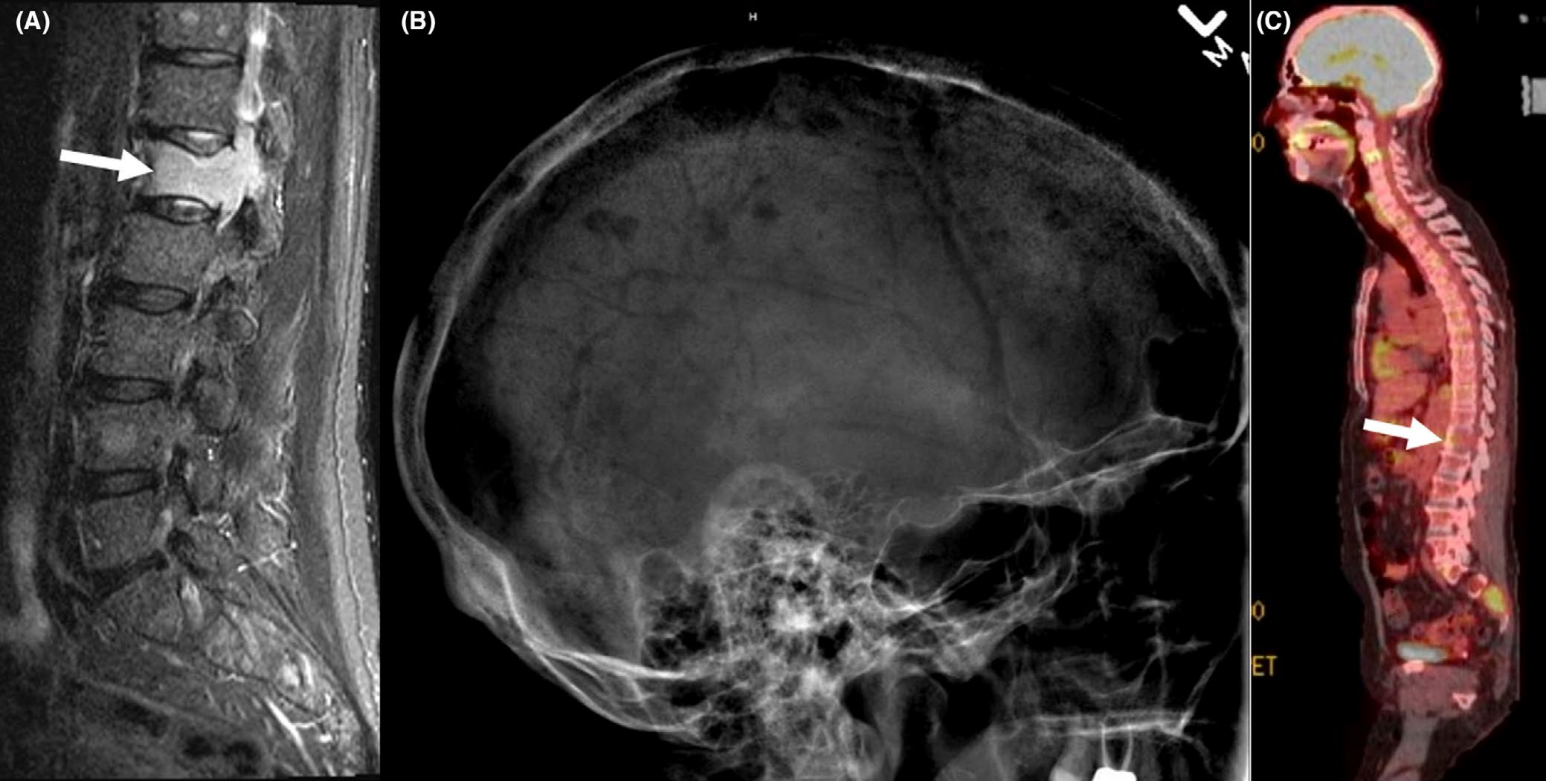
A, Sagittal fat suppressed T2 weighted MR image of the lumbar spine shows a pathologic fracture of the L1 vertebral body with bone marrow edema (arrow). Additional lesions are present in the T11 and L4 vertebral bodies. B, Lateral radiograph of the cranium shows multiple well defined “punched out” lytic lesions. C, Sagittal fusion image from PET/CT shows multiple lytic lesions in the spine with variable FDG uptake. A large lytic lesion is noted in the L1 vertebral body (arrow)

A year later (2017), the patient developed lower back pain. MRI of the spine showed pathological fracture of the L1 vertebral body with bone marrow edema; additional lesions were present in the T11 and L4 vertebral bodies (Figure 2A). A bone marrow biopsy showed 27% plasma cells (Figure 1C, D) with kappa light chain restriction; 24‐hour urine sample showed increase Bence Jones protein, Kappa/lambda 34 (normal: 0.26‐1.65), consistent with kappa light chain multiple myeloma. Lateral radiograph of the cranium showed multiple well defined “punched out” lytic lesion (Figure 2B). Whole body PET/CT showed multiple lytic lesions in the spine with variable FDG uptake; a large lytic lesion is noted in the L1 vertebral body (Figure 2C). FISH study on BM showed 8.5% with 13q14.3 deletion and no abnormality on chromosome 11; normal karyotype. Ibrutinib was discontinued and the patient was started on palliative radiation to the spine followed by RVD regimen^13^ (Lenalidomide [Revlimid^®^] 25 mg orally day 1‐14; Bortezomib [Velcade^®^ 1.3 mg/m^2^ subcutaneously on days 1,4,8,11; and Dexamethasone 40 mg orally on days 1,8,15). Treatment was repeated every 28 days. The patient received a total of five treatment cycles and achieved very Good Partial Response (vGPR). He was then evaluated for and underwent autologous stem cell transplantation (ASCT) on 9/27/2017. Patient recovered slowly. He was started on maintenance lenalidomide 10 mg daily around day 60 after transplant. His IEP continued to show suppressed immunoglobulins without paraprotein. The patient remained in remission during his last follow up two and a half years after his transplant.

## 3 DISCUSSION

Genetically, this patient presented with 2 malignant B‐cell clones, 11q‐ as the major clone, and 13q‐ as a minor clone. Clinically, morphologically and phenotypically, the disease manifested as CLL at initial diagnosis. This picture was maintained for the subsequent 3.5 years while the patient was under observation and was confirmed at time of disease progression. At that time, the patient was started on ibrutinib therapy and about a year later the picture completely changed to MM (bone disease, bone marrow plasmacytosis) while his CLL was well controlled. The common links between the two diseases in this patient were the monotypic immunoglobulin light chain restriction (both were kappa light chain‐expressing) and deletion of 13q (13q‐). At the time of myeloma diagnosis, FISH study showed 13q‐ abnormality only (8.5%). This finding indicates that ibrutinib therapy was effective in eliminating the 11q‐ clone but not the 13q‐ cells. Furthermore, the 13q‐ cells evolved into overt MM following the elimination of 11q‐ cells by ibrutinib therapy. What triggered evolution of the minor 13q‐ clone to overt myeloma is not clear. It is clear, however, that plasma cells were not visually increased in the bone marrow at time of CLL progression when FISH showed 13q‐ in 14.5% of nuclei analyzed. It is likely that additional genetic event(s), beyond detection by FISH, occurred that triggered evolution of the 13q‐ clone into overt myeloma. Several primary and secondary genetic events are known to be involved in myelomagenesis.^14^ Deletion of the long arm of chromosome 13 (13q‐) is reported in both CLL and MM.^15^ Several loci have been identified with 13q arm that contribute to the pathogenesis of disease. For example, 13q14 contains a “minimally deleted region” which was shown in CLL to harbor the tumor suppressor gene *DLEU2*/miR‐15a/16‐1.^16^ Deletion or mutation in these loci contributes to CLL pathogenesis. Similar analysis identified the same locus as a minimally deleted region in MM.^17^ However, it is not known whether DLEU2, miR‐15a, or miR‐16‐1 has tumor suppressor functions in MM similar to CLL. Additional areas of 13q deletion (13q34, in addition to 13q14) have been reported in multiple myeloma.^17^ Molecular studies of the CLL and MM cells in our patient could have provided additional insight into the molecular events associated with the clinical and morphological features; unfortunately, such studies were not performed.

Careful examination of clinical trials results indicates that ibrutinib, as monotherapy, has no significant activity in MM. In a phase 2 study published by Richardson et al, the highest dose of ibrutinib given alone to patients with relapsed/refractory MM did not produce responses although 33% of patients achieved “stable disease”.^18^ However, when the same dose (840 mg/day) was combined with weekly dose of dexamethasone (40 mg/week), 28% of the patients responded. There was no “dexamethasone alone” arm in the study to determine the role of ibrutinib (if any) in these responses since dexamethasone is known to be active in MM. Some evidence from preclinical studies indicates that ibrutinib may have potential beneficial therapeutic effects in this disease through inhibition of NF‐kB,^19^ inhibition of myeloma cell migration and bone disease,^20^ inhibition of osteoclast bone resorption,^21^ and enhancement of the proteasome inhibitor, bortezomib (Velcade) and lenalidomide (Revlimid) effect against myeloma cells through NF‐kB pathway.^22^ It is more appropriate, therefore, to consider ibrutinib as a potential “modulating” agent with secondary effects rather than a direct antimyeloma drug. Current trials incorporate ibrutinib in combination therapy regimens for MM. One such trial is sponsored by the Alliance Foundation Trials, LLC incorporating ibrutinib with lenalidomide and dexamethasone (NCT 03702725).

Lack of direct antimyeloma activity of ibrutinib should not be surprising given its known mechanism of action. The drug is a small molecule inhibitor of the B‐cell receptor (BCR) signaling pathway through covalent binding to, and irreversible inhibition of Bruton tyrosine kinase (BTK).^3^ In contrast to memory and other mature B cells, plasma cells do not depend on BCR for survival and proliferation. Surface BCR is, therefore, downregulated on plasma cells.^23^ Given these biological features, a drug like ibrutinib is not expected to exert antiproliferative or apoptosis effects against plasma cells of MM. Some preclinical studies suggested that BTK may be a therapeutic target for “stem cell‐like myeloma cells” that express elevated levels of BTK^24^ but so far this intriguing finding has not been supported by clinical studies.

One should note the limitation of our observation in this report that it is one patient and may not speak to the broad issue. It is possible that ibrutinib may prevent emergence of MM in a subset of CLL patients. However, a large number of patients with CLL on ibrutinib therapy monitored for prolonged period of time will need to be analyzed and reported. In the meantime, additional anecdotal reports like ours may provide an alternative definitive answer.

## CONFLICT OF INTEREST

None declared.

## AUTHOR CONTRIBUTION

AA: conceived and funded the project, and contributed to overall supervision, patient care, and manuscript writing and editing. HG: contributed to literature search, gathering of case report data, and assisted in manuscript writing. DS: provided patient care, patient and literature data analysis, and manuscript review and editing. SA: review and interpretation of imaging data, composed and provided legend for the radiology figure, and contributed to manuscript review and editing. MB: contributed to review and interpretation of pathology material, composed and provided legend for the pathology figure, and manuscript review and editing. ZK: contributed to patient care, and manuscript review and editing.

## Supporting information

Tab S1
