# Supplementary material for: Emergence of overt myeloma in a patient with chronic lymphocytic leukemia on ibrutinib therapy
Source: Clin Case Rep. 2020 Jul 15;8(9):1797–801. doi: 10.1002/ccr3.3019 (PMC7495781; doi:10.1002/ccr3.3019)
Supplement: Supplementary file 1 — Tab S1 [file CCR3-8-1797-s001.docx]

Supplementary table 1: Co-existing CLL/SLL and MM*

| Sequence of Diagnosis | # of patients (%) |
| --- | --- |
| Metachronous |  |
| - CLL/SLL followed by MM | 15 (53%) |
| - MM followed by CLL/SLL | 2 (8%) |
| Synchronous (CLL/SLL + MM) | 11 (39%) |
| Total | 28 (100%) |

*Data summarized from reference #1

CLL/SLL: chronic lymphocytic leukemia/small lymphocytic lymphoma

MM: multiple myeloma
